# Overview of patients’ cohorts in the French National rare disease registry

**DOI:** 10.1186/s13023-023-02725-2

**Published:** 2023-07-03

**Authors:** Thibaut Pichon, Claude Messiaen, Louis Soussand, Céline Angin, Arnaud Sandrin, Nabila Elarouci, Anne-Sophie Jannot, on behalf of the BNDMR infrastructure team

**Affiliations:** 1 French National Rare Disease Registry (BNDMR), Greater Paris University Hospitals (AP-HP), 33 bld de Picpus, Paris, 75012 France; 2 Université Paris Cité, INRIA Paris, Centre de Recherche des Cordeliers, HeKA, Inserm, Paris, France

## Abstract

In France, all patients followed by Rare Disease (RD) expert centers have to be registered in the National Rare Disease Registry (BNDMR). This database collects a minimum data set including diagnosis coded using the Orphanet nomenclature. Overall, 753,660 patients were recorded from 2007 to March 2022 including 493,740 with at least one rare disease diagnosis. Among these rare disease diagnoses, 1,300 diagnoses gathered between 10 and 70 patients and 792 gathered more than 70 patients, corresponding to more than one patient per million inhabitants. A total of 47 rare disease diagnoses with point prevalence or incidence reported in the literature below 1/1,000,000 have more than 70 patients in the BNDMR, suggesting larger BNDMR cohorts than expected from reported literature. As a conclusion, our national RD registry is a great resource to facilitate patients’ recruitment in clinical research and a better understanding of RD natural history and epidemiology.

In France, all patients followed by Rare Disease (RD) expert centers have to be registered in the National Rare Disease Registry (BNDMR). This database collects a minimum data set [1] including diagnosis coded using the Orphanet nomenclature (ORPHAcodes) [2]. Among 2200 rare disease expert centers in France, 97% have access to the information system allowing data collection for the BNDMR. We extracted in March 2022 all diagnoses and associated patients’ identification numbers from the BNDMR data warehouse [3], excluding fetuses and foreign residents. The different RD-specific cohorts were then built after patients’ deduplication between hospitals.

Overall, records from 753,660 patients were extracted including 547,346 with at least one ORPHAcode and 206,314 without registered diagnosis in the database (no appropriate ORPHAcode found by the clinician, patients in diagnostic impasse or wandering). Among patients with an ORPHAcode, those with recorded diagnoses considered as not rare in Europe (prevalence greater than 1/2000) or described by a term representing a group of disorders were excluded (N = 53,606). Then, aggregation of ORPHAcodes representing subtypes at main disorder level resulted in the identification of 493,740 remaining patients. There were 5,429 ORPHAcodes with at least one patient in BNDMR from the 6,387 available ORPHAcodes for disorders. 1,300 ORPHAcodes gathered between 10 and 70 patients and 792 gathered more than 70 patients, corresponding to more than one per million inhabitants. Count for each diagnosis ORPHAcode can be found at https://www.bndmr.fr/publications/nombre-de-cas-par-mr/. Diagnoses were then further classified in the different categories according to their Preferential parent in the Orphanet Classification. Figure 1 describes the proportion of the different categories in terms of diagnoses and patients.

**Fig. 1:**
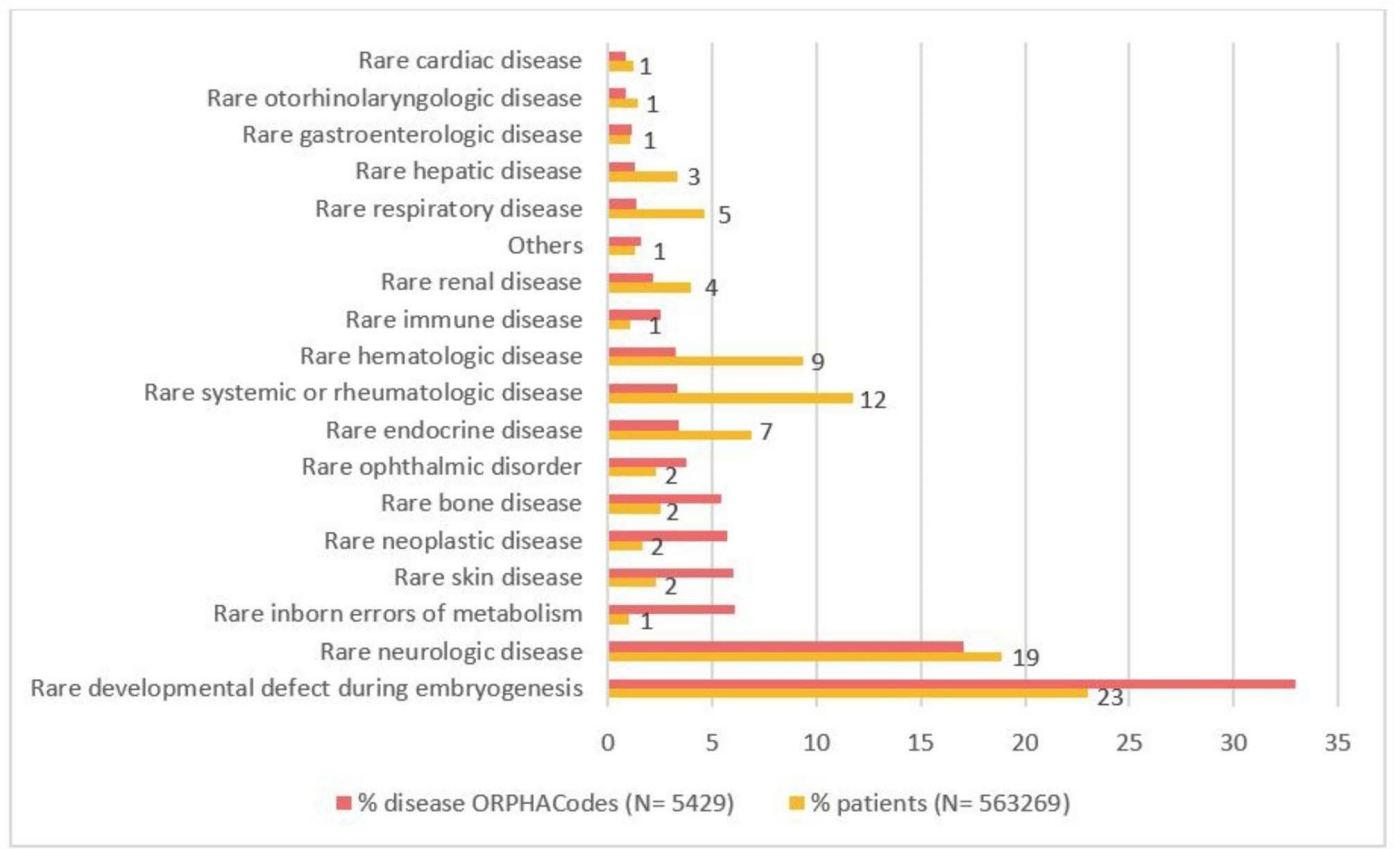
Number of diagnoses (ORPHAcodes) according to cohort size in the BNDMR, according to and documented epidemiological data from Orphanet for each diagnosis

We extracted from the Orphanet database the prevalence and the incidence reported worldwide or in Europe [2]. Figure 2 gives the number of ORPHAcodes according to BNDMR cohort size distributed by point prevalence or incidence and case reports documented by Orphanet. A total of 35 ORPHAcodes with point prevalence or incidence in Orphanet below 1/1,000,000 have more than 70 patients in the BNDMR, suggesting larger BNDMR cohorts than expected from reported literature. In particular, among these 35 BNDMR cohorts, nine include more than 200 patients: IL-10 related early onset inflammatory bowel disease, adult-onset cervical dystonia (DYT23 type), primary bilateral macronodular adrenal hyperplasia, neurofibromatosis type 3, sodium channelopathy-related small fibre neuropathy, Klippel-Trénaunay syndrome, cerebellar ataxia with neuropathy and vestibular areflexia syndrome (CANVAS), Coffin-Siris syndrome and Cloves syndrome. These figures need further investigations with RD expert centers to determine the French prevalence, as already demonstrated for the Fibrodysplasia ossificans progressiva [4]. For 228 diagnoses, the prevalence documented in Orphanet was higher than seen in the BNDMR data, probably due to a severity bias. Indeed, patients with mild forms of rare diseases are likely not to be followed in rare disease expert centers.

**Fig. 2:**
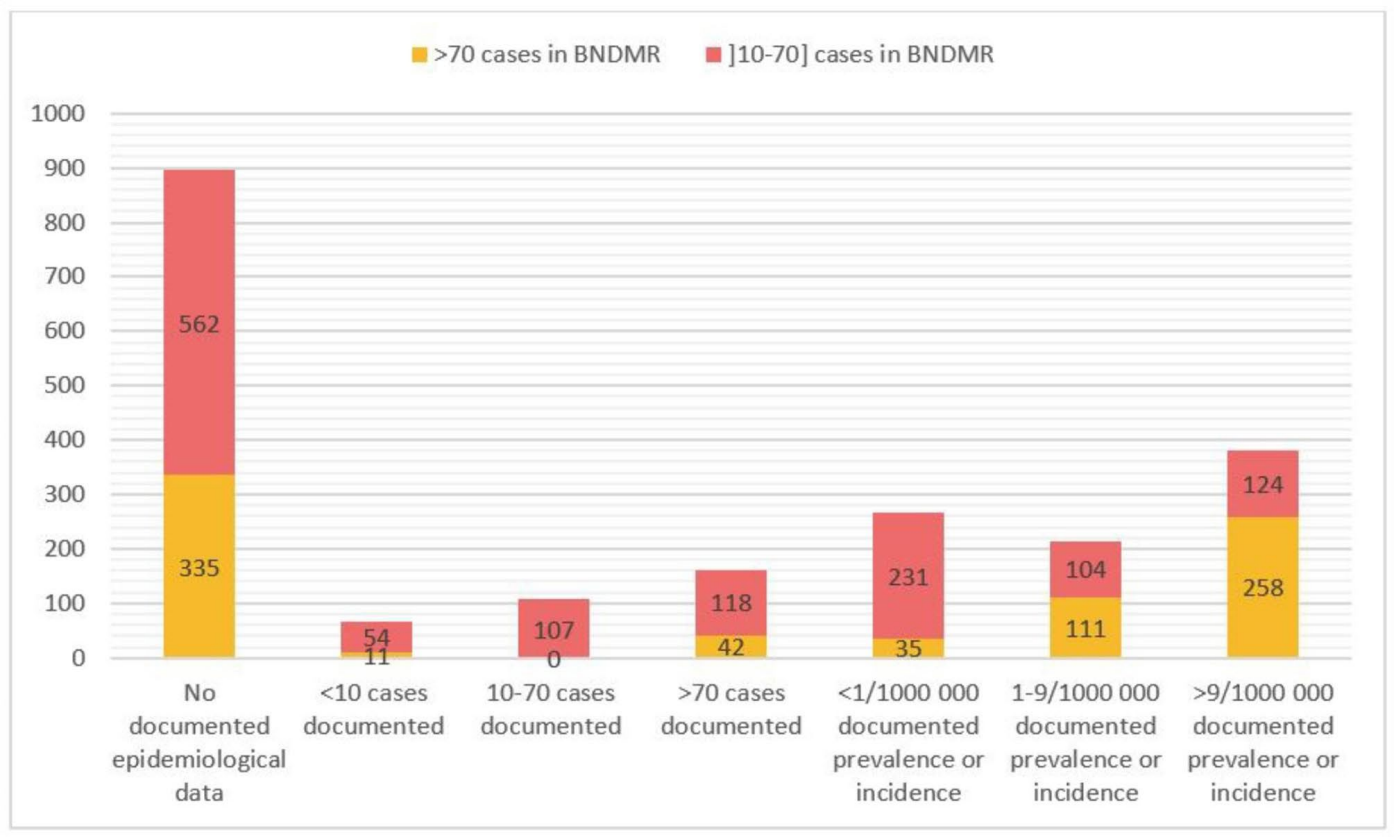
Proportion of the different categories in terms of diagnoses and patients. Diagnoses were classified in the different categories according to their Preferential parent in the Orphanet Classification

The idea sustained by the French Rare Disease Plan that identified expert centers enhance the appropriate RD patient orientation in care pathway is supported by those findings.

As a conclusion, a national RD registry implemented by expert centers is a valuable source to identify RD cohorts. It is a great resource to get insight of the number of patients with a given rare disease that can be recruited through the expert network for clinical research and a better understanding of RD natural history and epidemiology.

## Data Availability

The datasets analysed during the current study are available at https://www.bndmr.fr/publications/nombre-de-cas-par-mr/ and http://www.orphadata.org/
